# Metabolic Influence of Psychrophilic Diatoms on Travertines at the Huanglong Natural Scenic District of China

**DOI:** 10.3390/ijerph111213084

**Published:** 2014-12-16

**Authors:** Shiyong Sun, Faqin Dong, Hermann Ehrlich, Xueqing Zhao, Mingxue Liu, Qunwei Dai, Qiongfang Li, Dejun An, Hailiang Dong

**Affiliations:** 1 Department of Geological and Mineral Engineering, Key Laboratory of Solid Waste Treatment and Resource Recycle & Fundamental Science on Nuclear Waste and Environmental Security Laboratory, Southwest University of Science and Technology, Mianyang 621010, China; E-Mails: shysun@swust.edu.cn (S.S.); zxqch@sina.com (X.Z.); dragonlmx@126.com (M.L.); qw_dai@163.com (Q.D.); liqiongfang992605@126.com (Q.L.); 2 Institute of Experimental Physics, Technische Universität Bergakademie Freiberg, Freiberg 09599, Germany; E-Mail: hermann.ehrlich@physik.tu-freiberg.de; 3 Huanglong National Scenic Resort Administrative Bureau, Songpan 623300, China; E-Mail: andejun.618hl@126.com; 4 Department of Geology and Environmental Earth Science, Miami University, Oxford, OH 45056, USA; E-Mail: dongh@miamioh.edu

**Keywords:** psychrophilic diatom, metabolic interactions, travertine, biomineralization, Huanglong

## Abstract

Diatoms are a highly diversified group of algae that are widely distributed in aquatic ecosystems, and various species have different nutrient and temperature requirements for optimal growth. Here, we describe unusual psychrophilic diatoms of Cymbella in a travertine deposition environment in southwestern China in winter season. Travertine surfaces are colonized by these psychrophilic diatoms, which form biofilms of extracellular polysaccharide substances (EPS) with active metabolic activities in extremely cold conditions. The travertine in Huanglong, is a typical single crystalline calcite with anisotropic lattice distortions of unit cell parameters along axes of *a* and *c*, and this structure is suggestive of some level of metabolic mediation on mineralization. Near-edge X-ray absorption fine structure spectroscopy (NEXAFS) results further confirmed the occurrence of biogenic distortion of the crystal lattice of travertine calcite. Overall, our results imply that the metabolic influence of psychrophilic diatoms may be particularly important for promoting formation and dissolution of travertine in extremely cold environments of Huanglong. The EPS of psychrophilic diatoms will protect travertine from HCO_3_^−^ etching and provide template for forming travertine when water re-flowing, in warm season.

## 1. Introduction

Travertine is a form of carbonate deposit in mineral springs [1,2]. Evidence obtained from previous laboratory and field-based experiments suggests that bacteria (particularly, cyanobacteria), algae, fungi, and bryophytes play significant roles in carbonate deposition and contribute to the formation of microbial biofilms and mats [3,4,5,6]. In the geological record, microbial carbonates are found most extensively in marine, lacustrine, spring, cave, and soil environments [3], where microbial cells may participate in the process of carbonate deposition via cell surface interactions. Deposition can also be mediated metabolically by the secretion of extracellular polysaccharide substances, also known as EPS, which act as preferential sites for nucleation and localized templates that force mineralization on the surface of the EPS matrix or as inhibitors of carbonate formation depending on intrinsic conditions [4,7,8,9].

Despite considerable progress, the role of microbial activity in travertine formation remains subject to intense scientific controversy owing to difficulties in discriminating microbial regulated mineral precipitates from carbonate formed in other ways, such as by inorganic (e.g*.*, physical or chemical) precipitation. Inorganic mechanisms typically control mineralization in supersaturated conditions [10] and have been investigated using numerous analytical techniques. The structural refinement results by X-ray diffraction (XRD) of biogenic, geological, and synthetic calcite suggest that crystal lattice distortion is a strong indicator that biotic processes control (or significantly influence) precipitation of travertine, likely through co-precipitation or embedding of biomolecules in the crystal structure [11,12,13]. Furthermore, the formation of calcite by cyanobacteria has been investigated using synchrotron-based scanning transmission X-ray microscopy combined with near-edge X-ray absorption fine structure spectroscopy (STXM-NEXAFS) [8,9]. These previous investigations detected and characterized the relationships between calcifying surfaces (e.g*.*, on cell surfaces, within the extracellular polysaccharide substances (EPS)) upon which microbial mediated mineral nucleation and precipitation processes occur. Additionally, fluorescent microscopy observations of biopolymers that exhibit autofluorescence and the use of contrast agents specific to certain biopolymers [14,15,16] can directly identify their localization within biomineral structures. These results suggest that identifying the structural and chemical characteristics of biogenic carbonate should improve understanding of the contribution of microbial activity to travertine deposition.

In recent decades, the role of microbes in geothermal travertine deposits has garnered increasing attention, particularly in studies investigating the origins of life, owing to the similarities between the geochemical environments of hot spring systems, the early Earth, and other planets of the solar system [5,17,18,19]. Numerous experiments have shown that microbial activity can play a significant role in travertine deposition from thermal springs and that, in nature, multiple biotic and abiotic factors combine to influence travertine deposition [2,5,11,20]. Nevertheless, aqueous geochemistry data indicate that the formation of travertine under ambient conditions is controlled primarily by inorganic processes, with phototrophs, such as diatoms playing negligible roles [10,21]. In contrast, recent investigations have revealed that microbial activity is rather important for the formation of travertine [10].

It is well known that temperature is an important factor that affects the survival of living organisms. In particular, when water freezes to ice, living species are faced with major challenges in regards to maintenance of biological processes such as metabolism, development, reproduction, biomineralization, and skeletogenesis. Psychrophilic diatoms or cold-favorable diatoms can be regarded as one of the most extremophilic eukaryotes on our planet, which has ability to thrive at temperatures around the freezing point of water [22,23]. The Huanglong travertine deposits in southwestern China are well known for their unusual and diverse landscapes, which include spring-fed streams, waterfalls, pools, and shoals [24,25,26]. The results of previous studies have suggested that the algae community in Huanglong Valley is comprised of 80% *cyanophyta* (cyanobacteria), 15% *Bacillariophyta* (diatoms), and 5% others (*Xanthophyta*, *Chlorophyta*, and *Euglenophyta*) in summer section [27,28]. For almost half the year, these deposits are covered with snow and experience extremely cold environmental conditions (*i.e*., 0 to 4 °C), with an annual mean temperature of 1.1 °C [24,29]. However, investigations at Huanglong Valley have revealed that the travertine surfaces are colonized by abundant psychrophilic diatom genus of *Cymbella* in wintertime. Two species of *Cymbella cymbi formis* and *Cymbella gracilis* play a key role in dominating the assemblages of travertines under covering snow. In the present study, we adopt multidisciplinary techniques to identify the principles underlying the metabolic interactions between psychrophilic diatoms and travertine.

## 2. Materials and Methods

Huanglong was declared a World Heritage Site by United Nations Educational, Scientific and Cultural Organization (UNESCO) in 1992. The site of travertine deposition in Huanglong Valley is located in Songpan County, Sichuan Province, southwestern China (32°45ʹ N, 103°50ʹ E). The travertine in the core study area of Huanglong Valley formed in the late Pleistocene (~80 ka). The area of deposition has a total length of 3.6 km, it is 30–250 m wide and 9–20 m thick, and it lies at an altitude of 3100–3600 m [25,30,31]. No specific permissions were required to sample these locations for the field studies associated with the present work.

Representative samples falling within the first and second steps of Huanglong Valley were selected for characterization of the mineral crystalline phase and chemical composition. These samples were labeled as HLM-1 to HLM-6, and they represent the Jinshapudi shoal, Mingjing pool, Xishendong, Liantai fall, Feipuliuhui, and Yingbin pool, respectively.

Bubbled CO_2_ pre-treatments were conducted before scanning electron microscope (SEM) observations to investigate carbonate enrichment effects on travertines. Specifically, synthetic calcite and the travertine samples collected in the Jinshapudi shoal were treated with CO_2_. The whole process was conducted at room temperature under ambient conditions. In a typical experiment, 1 g of travertine was added to a reaction flask containing 100 mL ultrapure water. Then, CO_2_ was bubbled into the reaction flask and magnetically stirred for 30 min. The resulting samples were collected, washed several times with absolute ethanol, and dried in a vacuum oven at room temperature.

Specific regions of the polysaccharides in travertine samples were labeled by staining with the Calcofluor White (Sigma-Aldrich), which binds preferentially to the β-1,4-bonds of cellulose, chitin and other polysaccharides in EPS [14,16]. The fluorescence observations were conducted under a fluorescent microscope (DM2000, Leica). The morphologies of selected travertine samples were observed using an environmental scanning electron microscope (ESEM, XT30, and Philips) and SEM (S440, Leica).

Mineral phase characterization of travertine was conducted using an X-ray diffractometer (XRD X'Pert Pro, PANalytical) with monochromatized CuKa radiation and a LynxEye detector. The copper anode had tube voltage of 40 kV, a current of 40 mA, a 20–90° 2θ scanning range, a 0.02° step size, and scan speed of 0.3 s/step [32]. Qualitative crystallographic analysis was conducted by matching powder XRD patterns from the standard diffraction database, whereas quantitative analysis was performed using the FullProf Suite (February 2007 version) to allow structural refinement using the Rietveld method [33]. The STXM–NEXAFS experiments at the Ca L-edge were performed on the soft X-ray spectromicroscopy beamline (BL08UA) of the Shanghai Synchrotron Radiation Facility [34]. The samples for this analysis were resuspended in ethanol and dropped on a silicon nitride window (Shanghai NTI Co. Ltd, China) before being mounted onto the sample holder of the beamline and observed by soft X-ray spectromicroscopy. The NEXAFS characterization of calcium was recorded at energies around the Ca L_2, 3_ absorption edges (342–360 eV). Finally, the chemical composition of travertine was analyzed both qualitatively and quantitatively by time-of-flight secondary ion mass spectrometry (TOF-SIMS V, ION-TOF GmbH) and X-ray fluorescence analyses (XRF, PANalytical), respectively.

## 3. Results and Discussion

Travertine samples were collected from shoals, pool sediments, and travertine dam edges. The psychrophilic diatoms (particularly, two species *of C. cymbi formis and C. gracilis*) are especially dominant in the Jinshapudi sloping shoal. However, psychrophilic diatoms are not significant populations in warm season.

**Figure 1:**
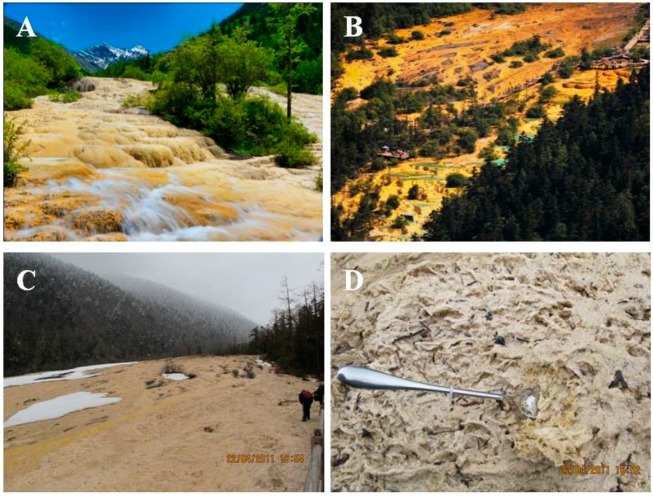
Photographs of the famous Jinshapudi sloping shoal. Panel (**A**) in July; (**B**) in September. Panel (**C**), in April. Panel (**D**), travertine samples collecting sites as shown in (C).

This shoal is about 1300 m long, and it has a maximum width of 125 m and a relative elevation of 116 m. The Jinshapudi shoal is thought to be one of the largest active travertine slopes worldwide [25]. In this shoal, the mixing of spring water and snowmelt forms a thin layer of fast-flowing shoal water from April/May to August (Figure 1A). However, this shoal flow is suspended from October to April when the shoal is covered with snow (Figure 1B,C). The travertine shoal surface is colonized by psychrophilic diatoms, which form a layer of yellow floccules and widespread mats at the stream bottom (Figure 1D).

**Figure 2:**
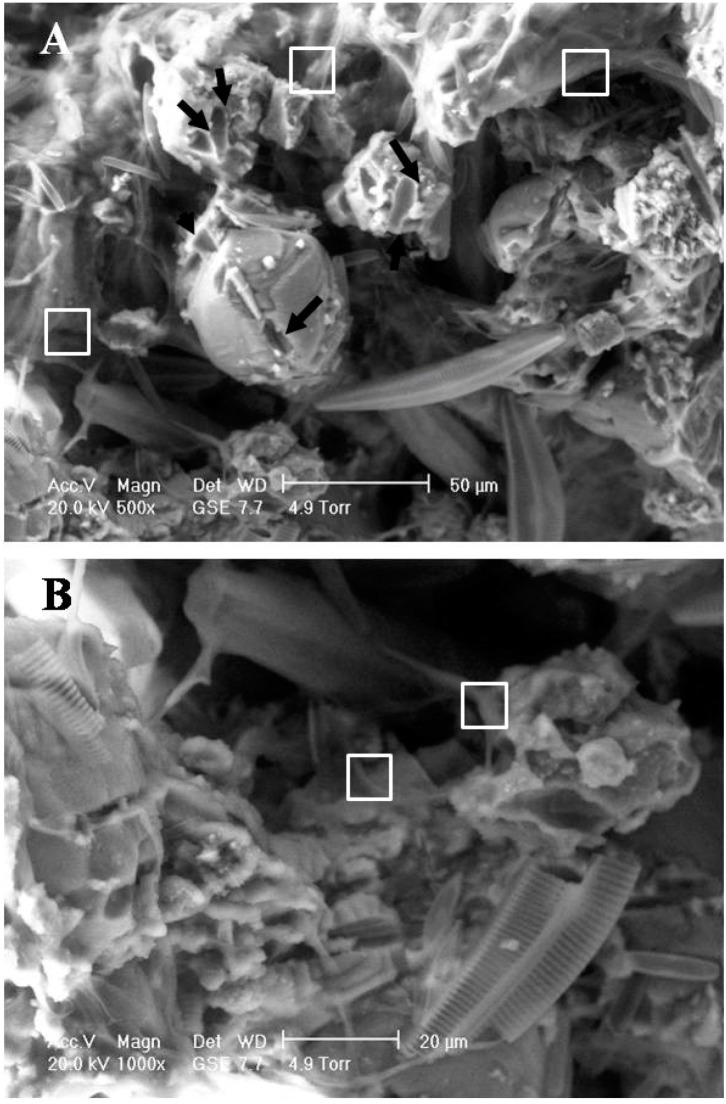
ESEM observation of a travertine sample, collected from the site shown in Figure 1D. Panels (**A**) and (**B**) show that the travertine surface was dominated by psychrophilic diatoms. Their metabolic activity has strong effects on the travertine surface as arrows indicated. Squares in Figure 2 indicate glutinous layers.

Observations demonstrated that numerous psychrophilic diatoms adhere to the travertine surface via the formation of a glutinous layer (Figure 2A). Particles observed in the travertine exhibited a wide size distribution ranging from sub-micron size to 50 μm (Figure 2B). We suggest that the metabolic activity of psychrophilic diatoms leads to erosion of the travertine particle surface, as suggested by the presence of numerous micro-grooves and holes (Figure 2).

**Figure 3:**
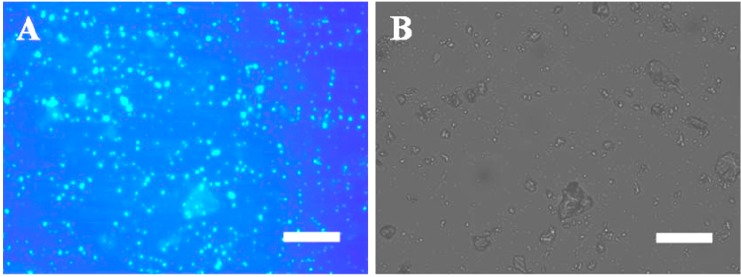
Photomicrographs showing travertine characterization by fluorescence microscopy which tavertine samples collecting at Jinshapudi sloping shoal in April. Panels (**A**) and (**B**) showing β-1, four-bond of polysaccharides labeled by Calcofluor white. Fluorescence image is shown in panel (A) and their blue colors indicate fluorescence signal. The bright field image is shown in panel (B). Scale bar: 100 μm.

The presence of EPS was also identified using Calcofluor White staining (Figure 3). Abundant EPS were found to be embedded within travertine particles (Figure 3A) and may have originated from microbes. Figure 3B presents a bright-field microscopy image of travertine particles with abundant psychrophilic diatoms associated with their surfaces. It is well known that EPS cannot be derived from inorganic CaCO_3_; thus, it is likely that the EPS were sourced primarily from the diatoms (Figure 2 and Figure 3).

Our XRD results demonstrate that the mineral phase of travertine is highly consistent with the calcite standard. Further quantitative crystallographic interpretation by structural refinement found that the unit cell of calcite from travertine exhibits stretching along its axes of a and c (Table 1). Conversely, calcite that is mineralized via inorganic control mechanisms has been shown to exhibit compression of the calcite unit cell c-axis [11,13]. Therefore, the XRD results from the Huanglong travertine are consistent with a mechanism proposed previously for the formation of calcite under strong biogenic influence [11,12,13].

**Figure 4:**
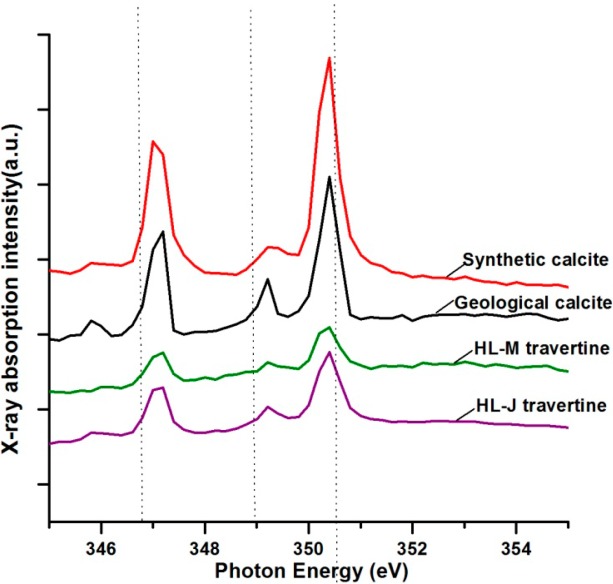
NEXAFS spectra measurement of Ca L_2, 3_ absorption edges of travertine and control calcite. Spectrum of HL-M travertine represents sample of HLM-2. Spectrum of HL-J travertine represents sample of HLM-1.

Near-edge X-ray absorption fine structure spectroscopy measurements have been used to determine the average oxidation state, the coordination environment, and subtle geometrical distortions of absorbing elements in samples [35]. In the present study, the NEXAFS spectra of travertine at the Ca L_2, 3_ edges were acquired with high spectral (~0.1 eV) and spatial (~50 nm) resolutions. We found the characteristic four-peak spectra of travertine to be similar to those of reference calcites, such as synthetic and geological calcites, although there were some disparities in the intensities of peaks (Figure 4). Moreover, the intensities of the synthetic and geological calcite signals were typically greater than that of the travertine from Huanglong, which provides further evidence of the occurrence of biogenic distortion in these crystal lattices. This crystal distortion was also confirmed through XRD structural refinements (Table 1).

**Table 1:** Results of structural refinements using Rietveld methods by quantitative X-ray diffraction.

Sample Name	a/Å	c/Å
HLM-1	4.99014（5）	17.0705（2）
HLM-2	4.98940（5）	17.0637（2）
HLM-3	4.99035（6）	17.0661（3）
HLM-4	4.99022（8）	17.0688（4）
HLM-5	4.99063（8）	17.0678（3）
HLM-6	4.98887（6）	17.0647（2）
Ref. a [11]	4.9868(2)	17.064(1)
Ref. b [13]	4.98879(8)	17.05940(2)

**Figure 5:**
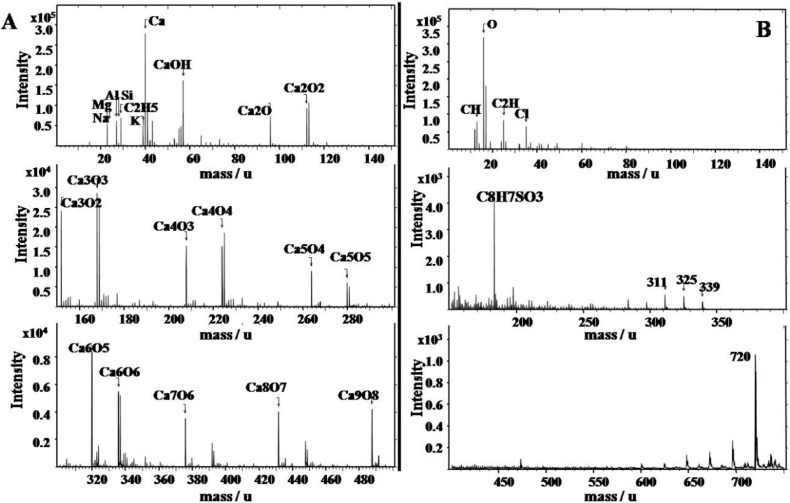
Qualitative chemical composition analysis by Time of Flight Secondary Ion Mass Spectrometry. Column (**A**), the positively charged ions. Column (**B**), the negative charged ions.

The qualitative chemical composition analysis by TOF–SIMS were carried out for spectroscopy identifying molecular (inorganic and organic) and elemental (positive and negative) specieson resolution of 0.00× amu with the concentrations of 0 to 10,000 amu. Travertine at Huanglong contains inorganic elements such as Ca, Mg, Si, Na, K, and Al in addition to organic macromolecules (Figure 5). Moreover, it is very interesting that organic sulfur is present in the form of C_8_H_7_SO_3_. The travertine deposition environment in Huanglong can be characterized as a low temperature (annual mean temperature of 1.1 °C) spring system, unlike containing high contents of inorganic sulfates of travertines in hot springs such as those at Yellowstone National Park of USA [5]. Therefore, travertine containing organic sulfur may be derived from microbial organisms, such that inorganic sulfates may not participate in travertine deposition [11]. According to our quantitative analysis of the chemical composition, CaCO_3_ is the main component of the Huanglong travertine, with 54% in the form of CaO. Furthermore, travertine samples collected in the Jinshapudi shoal exhibit much higher silicon contents and display significant loss on ignition (LOI) (Table 2), yet the samples for LOI analyses were prepared carefully for reducing water content. Therefore, it is reasonable to assume that the higher LOI was not derived from the water in the travertine; rather, it likely originated mainly from organic components of the psychrophilic diatoms within the travertine. The results of chemical compositions analysis indicate that travertine deposition in Huanglong both enrichment of calcium and silicon by metabolic interactions between psychrophilic diatoms and travertines.

**Figure 6:**
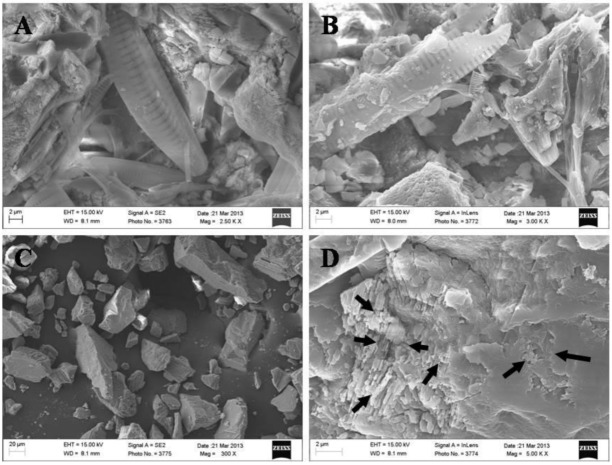
SEM micrographs of CO_2_ pretreated travertine sample and synthetic calcite. Panels (**A**), (**B**) show microscopy pictures of sample collected at Jinshapudi shoal. Panels (**C**), (**D**) show microscopy pictures of reference sample of synthetic calcite. Arrows in panel (D) show etching effects on synthetic calcite.

To estimate carbonate enrichment effects for travertine after snow melting and restoration of fast-flowing shoals, travertine samples and synthetic calcite were pretreated with bubbled carbon dioxide before SEM observations. The results demonstrate that the travertine surface was covered by EPS layers of psychrophilic diatoms (Figure 6A,B). These EPS layers appeared to protect the travertine from dissolution by CO_2_ etching (Figure 6A,B), whereas small particles of synthetic calcite were dissolved by this CO_2_ treatment (Figure 6C) and the surfaces of larger particles of synthetic calcite exhibited strong etching effects (Figure 6D).

Travertine biotic deposition in hot springs is typically mediated by biogenic sulfide bacteria [4,5,6]. In particular, sulfur-containing biomolecules are inserted into the calcite crystal lattice, which causes it to become distorted [11]. However, the microbial community in the Huanglong cold spring differs from that in hot springs, such as those at Yellowstone National Park in Wyoming, USA [24,25,26,27,28]. In such hot springs, deposition is determined by differences in temperature and the geochemical environment. Therefore, it should be expected that the microbial activity involved in the deposition of travertine in such springs is different from that in the cold spring environment of Huanglong.

Photosynthesis-induced carbonate precipitation (PCP) does not occur under all environmental conditions and suitable ambient water chemistry conditions are required for biologically induced mineralization; thus, aquatic phototrophs do not always calcify [7,10]. Therefore, PCP of travertine is found primarily in stationary pools, which provide conditions that are more favorable for phototroph growth. However, the famous travertine landscape of Huanglong is located within the fast-flowing Jinshapudi sloping shoal (Figure 1) [25,29]. In this environment, psychrophilic diatoms seem to be primarily responsible for the nucleation and localization of mineral deposition. Further evidence for the biogenic deposition of travertine in Huanglong was found based on examination of the EPS in samples from the study area. EPS of psychrophilic diatoms from Huanglong appear to control travertine deposition directly, forming patterns that do not normally develop within solutions that are highly saturated with dissolved Ca^2+^ and carbonate species. We compared the specimens from Huanglong to pure mineral specimens that we treated with CO_2_ in the laboratory; this simulates the suspended spring in the valley, which contains high concentrations of HCO_3_^−^ during springtime when the water flow is fast. This fast-flowing water may also dissolve travertine particles that were deposited previously. The simulation experiments show that travertine from Huanglong may be protected from HCO_3_^−^ etching by EPS layers produced by psychrophilic diatoms, whereas synthetic calcite was clearly affected by etching (Figure 6). Therefore, the metabolic interaction of psychrophilic diatoms in travertine deposition may be important for the formation of the Huanglong travertine deposits and the widespread growth of psychrophilic diatoms.

**Table 2:** Quantitative chemical composition analysis by X-ray fluorescence analysis (in % by wt.).

Sample Name	SiO_2_ %	Al_2_O_3_ %	Fe_2_O_3_ %	MgO %	CaO %	Na_2_O %	K_2_O %	MnO %	TiO_2_ %	P_2_O_5_ %	LOI %
HLM-1	0.826	0.02	<0.01	0.309	54.47	<0.01	<0.01	<0.004	0.006	0.005	44.13
HLM-2	0.604	0.111	<0.01	0.414	54.57	0.014	0.016	<0.004	0.011	0.008	44.18
HLM-3	0.74	0.06	<0.01	0.391	54.34	<0.01	0.018	<0.004	0.01	0.01	44.16
HLM-4	0.506	0.088	<0.01	0.358	55.24	0.016	0.011	<0.004	<0.006	0.005	43.72
HLM-5	0.345	0.022	<0.01	0.342	55.17	<0.01	0.01	<0.004	0.008	0.009	43.85
HLM-6	0.376	0.07	<0.01	0.346	55.5	<0.01	0.014	<0.004	0.011	0.007	43.59

## 4. Conclusions

In the presented study, we investigated the mineral characteristics of travertine in Huanglong Valley. The results show that psychrophilic diatoms of *C. cymbi formis* and *C. gracilis* are likely dominant microbes in such extremely cold ecosystem. In particular, our results indicate that psychrophilic diatoms play a complicate role in the formation and dissolution of travertine through metabolic activities. Psychrophilic diatoms traps travertine particles and play a positive role in precipitating travertine when the water flows slowly under low temperature before freezing. As far as psychrophilic diatoms under snow covered, psychrophilic diatoms may participate interactions with travertine in the manner of metabolic inducing dissolving calcium from travertine particles. The EPS layers which mediated from psychrophilic diatoms will protect travertine from HCO_3_^−^ etching and provide template for forming travertine when water re-flowing in warm season. The characteristics of Huanglong travertine, to some extent, are similar to those of calcite produced by bio-mediating mechanisms as described previously.
